# Synergy of Lewis and Brønsted acids on catalytic hydrothermal decomposition of carbohydrates and corncob acid hydrolysis residues to 5-hydroxymethylfurfural

**DOI:** 10.1038/srep40908

**Published:** 2017-01-13

**Authors:** Chao Wang, Liming Zhang, Tian Zhou, Jiachuan Chen, Feng Xu

**Affiliations:** 1 Beijing Key Laboratory of Lignocellulosic Chemistry, Beijing Forestry University, Beijing, 100083, China; 2 Shandong Key Laboratory of Pulping and Papermaking Engineering, Qilu University of Technology, Jinan, 250353, China

## Abstract

5-hydroxymethylfurfural (HMF) is an important platform molecule in the synthesis of various chemicals and materials. Herein, we reported a simple and effective dehydration of glucose-based carbohydrates to HMF in a biphasic system containing cyclopentyl methyl ether as the organic phase and AlCl_3_ with minute amounts of HCl as co-catalysts. The results showed that the mixed catalysts had a positive synergistic catalytic effect on glucose conversion to HMF compared with single AlCl_3_ or HCl catalyst. For glucose, the highest HMF yield of 54.5% was achieved at 175 °C for 20 min. More importantly, the optimal catalytic system was so efficient that it achieved one of the highest reported yields of HMF (30.5%) directly from corncob acid hydrolysis residues. Thus, the catalytic system can become a promising route for effective utilization of biomass in future biorefineries.

Increasing concerns for fossil fuels depletion and environmental pollution have prompted search for sustainable alternative sources for energy and chemicals. Renewable biomass, such as agricultural wastes, forestry residues and energy crops is abundant and environmentally compatible alternatives to fossil sources for liquid fuels and chemicals on a large scale^1^. Corncob acid hydrolysis residues (CAHR) is a typical lignocellulosic waste, being generated during the production of xylose by converting the hemicellulose component in the presence of mineral acids. CAHR is commonly regarded as low value material, which needs to be treated urgently^2^. Actually, CAHR is rich in cellulose and its derived hexoses^3^, which serves as important platform for generation of valuable products such as ethanol^4^, 5-hydroxymethylfurfural (HMF), and levulinic acid (LA)^3^.

HMF holds great promise as a primary building block of biorefinery^5^ since it can be catalytically upgraded as a starting material with high selectivity to drop-in chemicals like dimethylfuran^6^, caprolactone^7^, and 5-furandicarboxylic acid^8^. However, efficient production of HMF from glucose using less corrosive acids and minimizing formation of undesirable by-products is a great challenge^9^,^10^,^11^,^12^. Glucose which only contains 1% of furanose tautomers was converted to HMF less efficiently than fructose which contains 21.5% of furanose tautomers. An effectual reaction system that overcomes the challenge in the dehydration of glucose to HMF is a key issue in selectively isomerizing glucose to fructose in tandem with the transformation reaction.

Selecting an efficient catalyst for glucose isomerization has a significant impact on glucose conversion to HMF. Isomerase and Lewis acids are catalysts commonly used for glucose isomerization. Glucose isomerases are able to typically industrially produce fructose from glucose^13^. Simeonov *et al*.^14^ achieved success with enzymatic isomerization of glucose to fructose in the first reactor, followed by Brønsted acid catalyzed fructose dehydration to HMF in the second reactor. Huang *et al*.^15^ further developed a tandem catalysis system, composed of a thermophilic glucose isomerase for glucose isomerization to fructose and a solid acid catalyst for fructose dehydration to HMF with 30% yield. However, the limitations of these methods were high cost of enzymes and high sensitivity of enzymatic reaction under the operating conditions. In comparison, Lewis acids not only offer highly selective glucose isomerization to fructose, but can also tolerate Brønsted acidity and high temperature. In our previous work, HMF yield exceeding 50% was achieved in a one-pot reactor, containing Lewis acidic Sn-Beta and Brønsted acidic HCl^16^. Other studies investigated advanced tandem heterogeneous Lewis/Brønsted acid catalysts for accelerating the degradation of sugars, such as dealuminated zeolites^17^, activated carbons^18^ solid superacid SO_4_^2−^/ZrO_2_-Al_2_O_3_^19^,^20^. Heterogeneous catalysts have been widely employed during HMF production because of their easy separation. However, solid acids catalysts present additional challenges, such as complexity and high-cost of the preparation process as well as catalysts deactivation due to the deposition of humins on the surface. Recent studies have shown that homogeneous metal chlorides (CrCl_3_/HCl) can also effectively catalyze isomerization and dehydration^21^,^22^. Comparison of CrCl_3_ with AlCl_3_ clearly shows that the latter is a safer choice while possessing comparable catalytic properties^12^. A related work on AlCl_3_-catalyzed conversion of carbohydrates to HMF was reported by Hu and co-workers^23^. They found that glucose could be effectively converted to HMF (61%) in THF-H_2_O biphasic system. However, moderate Brønsted acids such as mineral acids used in the present of AlCl_3_ for glucose conversions are meaningful for reducing the large amounts of AlCl_3_ (40 mol% equality to carbohydrate units), so the AlCl_3_-catalytic conversion of carbohydrate is still needed to investigate in detail.

Solvents are also expected to play an important role on HMF formation. Water is an ideal green solvent for transformation of sugars, but the poor stability and high solubility of HMF in water results in low HMF selectivity and difficulty in HMF isolation, which would limit industrial scale application of a simple aqueous phase. Ionic liquids (ILs) are efficient media for glucose dehydration to HMF, and ILs have been widely investigated in the production of HMF from biomass. Zhao *et al*.^24^ reported chromium catalysts in [EMIM]Cl, enable synthesis HMF from glucose in good yield (68–70%). However, there are some potential limitations for large scale production of HMF using ILs. ILs is generally expensive and their stability at high temperature is also a concern. Furthermore, because of high solubility of HMF in ILs isolation of HMF poses major technological challenge, especially for large scale production. For facilitating isolation of targeted products, biphasic reaction systems are advantageous in terms of their ability to extract HMF to the organic phase before it undergoes further dehydration to organic acids or reacts with glucose and/or other HMF molecules, forming humins. The effective biphasic system mainly consists H_2_O and an extracting organic solvent, such as toluene, 2-butanol, methyl isobutyl ketone^25^, tetrahydrofuran (THF)^26^, *sec*-butylphenol^27^ which can improve HMF yields via extracting the reactive HMF product to the organic solvent, decreasing the possibility of side reactions. Molina *et al*.^28^ reported on dehydration of xylose in water/cyclopentyl methyl ether (CPME), which effectively inhibited generation of undesired products, resulting in 100% yield of furfural at 170 °C using H_2_SO_4_. Recently, Guenic *et al*.^29^ achieved 74% yields of furfural in water/CPME, using FeCl_3_ as catalyst. CPME has become available in commercial quantities since November 2005^30^. It presents several advantageous features of a potential solvent, such as low boiling point, low formation of peroxides, low price, relative stability under acidic and basic conditions high hydrophobicity and its limited miscibility, which allows easy separation and recovery from water. CPME is a competitive alternative to other ethereal solvents, such as THF, 2-methyl tetrahydrofuran (2-MeTHF), and other solvents mentioned above. These advantages make CPME a promising and environmentally compatible solvent for catalytic production of HMF from plant biomass.

The object of this work is to achieve the high efficiency production of HMF directly through glucose rather than fructose with co-catalysts of AlCl_3_ and HCl in the CPME/H_2_O biphasic system. The study investigated the effect of reaction conditions, such as temperature, time, CPME loading, and the influence of NaCl addition on HMF yield. Furthermore, our optimal conditions are employed to proceed transformation of corncob acid hydrolysis residues (CAHR) into HMF.

## Results and Discussion

### Performance of catalysts for converting glucose into HMF

**Figure 1:**
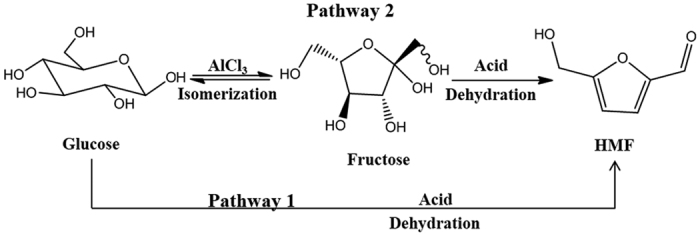
Conversion of glucose to HMF by two pathways with AlCl_3_/HCl catalysts.

In this section, the effect of different catalyst types such as mineral acid (HCl), metal chlorides (AlCl_3_), and the combination (AlCl_3_/HCl) was studied on the dehydration of glucose at 175 °C for 20 min in the biphasic system (CPME-H_2_O, v/v). When Brønsted acid HCl (pH = 2.50) is introduced into the biphasic system, the poor yield of HMF is only 4.5% at 15.5% of glucose conversion (entry 1, Table 1). Fructose was not detected in HCl-catalytic reaction alone, indicating that no glucose isomerization to fructose occurred in Brønsted acid catalytic system. This is because the conversion of glucose to HMF by HCl as catalysts is mostly to proceed through direction dehydration of glucose by pathway 1 (Fig. 1). In the presence of Lewis acid catalyst (AlCl_3_), the yield of HMF was 46%, and near absolute conversion of glucose was achieved in the system, observing the occurrence of fructose. In previous reports^31^,^32^, it was found that the main active species in glucose-to-fructose isomerization reaction was most possibly the hydrolyzed aluminum species [Al(OH)_2_(aq)]^+^. Combined with Lewis acid and Brønsted acid, HMF yield was increased to 54.5%, and this improvement might be attributable to decreased pH (pH = 3.37 to pH = 2.50). The results revealed that the addition of HCl as a co-catalyst to AlCl_3_ can to some extent improve the yield and the selectivity of HMF. Fringuelli *et al*.^33^ found that the pH of the solution plays a critical role in adjusting the Lewis acidity of metal halides in aqueous phase. Indeed, an increase in acidity also expedites fructose conversion to HMF, since not only does the addition of HCl increase the content of Brønsted acid sites, but also possibly adjust the equilibrium of Al^3+^ aqua/hydroxo complexes 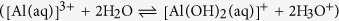. As showed in Table 1, when certain amount of HCl (n_HCl_/n_AlCl3_ = 0.11) and AlCl_3_ mixtures were used as the acid catalyst, the concentration of [Al(OH)_2_(aq)]^+^ species was lower than that with no HCl added^31^, which resulted in fructose yield decreasing. Although the fructose yield was slightly decreased by adding HCl, the addition of HCl facilitated the dehydration of fructose to HMF. The one-pot reactor adjusted the equilibrium-limited glucose to fructose isomerization to high conversion by means of dehydrating fructose to HMF thus overcoming the slow glucose conversion to HMF via Brønsted acid catalysis alone. Swift *et al*.^22^ also investigated that catalytic systems which contain both Lewis acidic sites and Brønsted acidic sites are more beneficial for HMF production than Lewis or Brønsted acidic catalysts alone.

The LA produced in the three above catalytic systems were also shown in Table 1. Only small amount of LA was formed, which also be suppressed by the introduction of organic extracting solvent according to previous literatures^16^,^34^. Additionally, less insoluble solid humins were observed when the addition of HCl is conducted, inferring that the formation of humins is attributed to Lewis acid sites. This result is also corroborated by the fact that the humins is probably generated by the cross-polymerization of intermediate and HMF^35^.

### Effect of the ratio of CPME to water on the conversion of glucose

**Figure 2:**
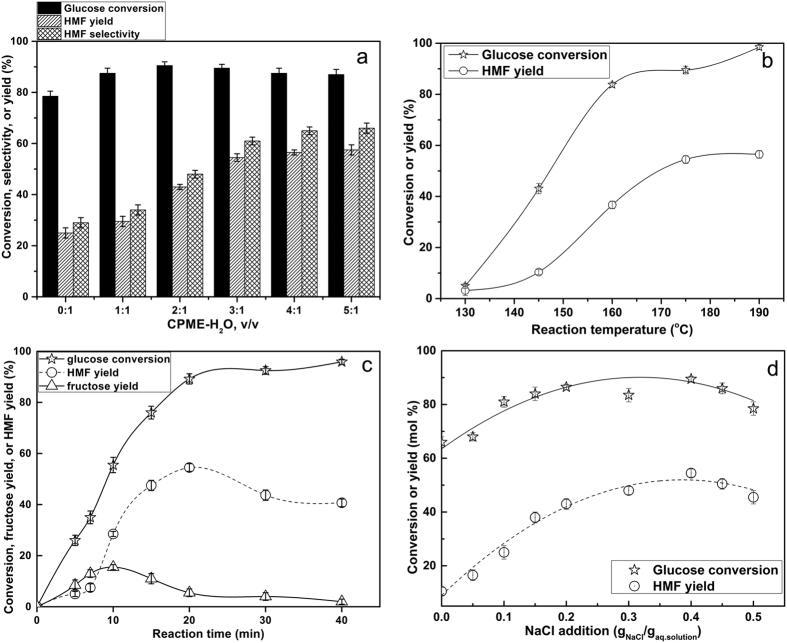
Effect of CPME-H_2_O ratio, reaction temperature, reaction time and NaCl concentration on dehydration of glucose to HMF. Reaction conditions: 10 wt% glucose in water (0.55 mmol), n (AlCl_3_) = 0.025 mmol, pH = 2.50. (**a**) NaCl/aqueous phase mass ratio of 0.4, reaction temperature of 175 °C, reaction time of 20 min. (**b**) NaCl/aqueous phase mass ratio of 0.4, CPME to water volume ratio of 3.0, reaction time of 20 min. (**c**) NaCl/aqueous phase mass ratio of 0.4, CPME to water volume ratio of 3.0, reaction temperature of 175 °C. (**d**) CPME to water volume ratio of 3.0, reaction temperature of 175 °C, reaction time of 20 min.

**Figure 3:**
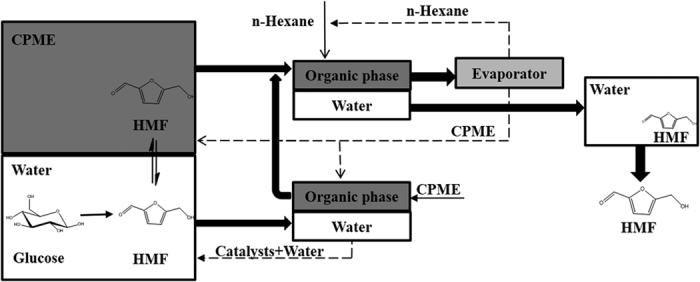
Dehydration process of glucose to HMF in the biphasic system CPME-H_2_O.

We conducted dehydration experiments with glucose, the least reactive but most abundant monosaccharide, by various ratio of CPME to water, with the goal of maximizing HMF yield and selectivity. Figure 2a showed the effects of various ratio of CPME to water. In water-only system, the HMF yield and the reaction selectivity to HMF were low (24.0% and 30.5%, respectively) while the glucose conversion was 78.5%. Thus, as can be expected, many undesirable reactions took place, and even water-insoluble solid products were formed without the organic solvent CPME^16^. The addition of CPME did not increase glucose conversion significantly, but the use of a CPME-H_2_O biphasic system selectively enhanced the production of HMF by the transfer of HMF to the organic phase after its formation in the aqueous solution. When increasing CPME ratio from 0% to 75%, the yield and selectivity of HMF increased to 54.5% and 61.0%, respectively. Moreover, the glucose dehydration process adjusts the equilibrium of reaction towards formation of HMF because CPME plays the role of storage for HMF (Fig. 3). With the addition of greater concentrations of CPME in the reactor, the yields of targeted products slightly increased. Additionally, when the volume ratio of CPME to water exceeds 3:1, the glucose conversion was decreased. This phenomenon might be explained by the several competing properties between water and CPME. CPME is a dipolar aprotic solvent with alkaline character, which can affect acid strength of the reaction mixture. Hence optimization of CPME concentration is important for achieving maximum HMF yield. CPME to H_2_O ratio of 3 was chosen for further experiments. Although HMF yield and HMF selectivity may slightly increase with larger CPME concentrations, excessive CPME concentrations are likely to be unfavorable to process competitiveness due to increased recovery costs, heating requirements, and reduced solids loading.

### Conversion of glucose into HMF in different reaction temperature and time

Reaction temperature and reaction time are two important factors which greatly affect the dehydration of glucose (Fig. 2b and c). To evaluate how the reaction temperature affected the production of HMF, the treatments were performed keeping all other parameters unchanged at same reaction conditions. Temperatures range of 130–190 °C was tested in order to determine the optimal reaction temperature (Fig. 2b). Glucose conversion increased sharply as the reaction temperature was raised from 130 °C to 160 °C for 20 min, as the reaction process was accelerated at higher temperatures. Over the same period the glucose conversion was 89.5% at 175 °C. At 190 °C glucose was fully converted. With increasing temperature, the HMF yield sharply increased and the maximum value (54.5%) was reached at 175 °C. The yield of HMF did not significantly increase at the higher temperature of 190 °C. These results demonstrated that the optimal temperature in the catalytic conversion of glucose to HMF was 175 °C.

To further explore glucose conversion behavior and HMF yield, the reaction time was varied from 5 to 40 min at the operating temperature of 175 °C and autogenous pressure (Fig. 2c). Fructose was detected as a dominant intermediate which reached a maximum concentration after 10 min. As the reaction time increased, fructose was consumed with concomitant formation of HMF. The HMF yield reached a maximum value of 54.5% after 20 min. These results clearly demonstrated that conversion of glucose was a typical tandem process. After 20 min, the yield of HMF started to decrease. Indeed, similar to the previous literature, the stability of HMF at high temperature for long time was low and HMF was quickly converted to degradation products^36^.

### Effect of different NaCl addition on the conversion of glucose

Since addition of salts to aqueous solutions improves the thermodynamics of solute extraction from aqueous phases to organic phases^37^, we studied the effect of NaCl addition on the HMF formation rate (Fig. 2d). Based on earlier work^25^, NaCl was selected as the additive, which showed that this salt is an optimal and cheap additive for the dehydration of glucose in a biphasic system. In this reaction, the glucose conversion and the yield of HMF increased as the NaCl concentration increased. It is apparent from the data provided (Fig. 2d) that addition of NaCl up to a certain value (about 0.4 g NaCl/g aqueous solution) significantly promoted both glucose conversion and HMF yields. The explanation offered is that NaCl as an additive in aqueous solution can improve the partitioning of HMF into CPME phase by salting-out effect, whereby electrolytes alter the intermolecular bonding interactions between liquid components, decreasing the mutual solubility of the aqueous and organic phase^38^. The observations presented of glucose conversion to HMF in a biphasic system catalyzed by AlCl_3_/HCl in the aqueous phase are consistent with those found for glucose dehydration in a water/THF biphasic system catalyzed by Sn-Beta zeolite and HCl^16^.

### Reusability of the catalysts

**Figure 4:**
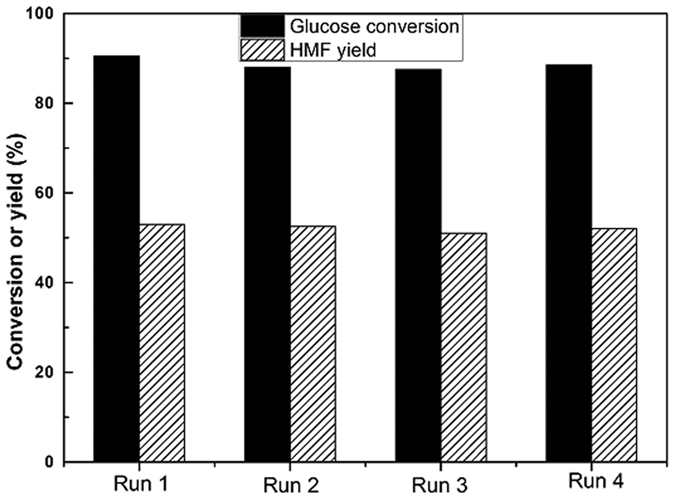
Reusability of the aqueous phase containing catalysts and NaCl for glucose dehydration to HMF. Reaction conditions: 10 wt% glucose in water (0.55 mmol), n (AlCl_3_) = 0.025 mmol, pH = 2.5, NaCl/aqueous phase mass ratio of 0.4, CPME to water volume ratio of 3, reaction temperature of 175 °C, reaction time of 20 min.

In order to demonstrate the effectiveness of our homogeneous catalytic process, series of recycling experiments about the acidic aqueous layer were performed using the conditions we had optimized. In a typical catalytic run, the reaction medium was separated and the organic CPME phase was removed by extraction (Fig. 3). Then, fresh glucose and CPME was added in the recycled aqueous phase without adding fresh catalysts. Four consecutive runs were performed with addition of a fresh substrate and organic CPME phase. The results (Fig. 4) indicate that no significant decreases in glucose conversion and HMF yields were observed.

The boiling points of CPME and HMF are alike, making it difficult to separate HMF from the solvent by distillation. Therefore, an alternative method is conducted by means of extracting HMF into a kind of solvent for lowing boiling point, such as water. In series of experiments, contacting an organic solution of 0.1 wt% HMF in CPME with only water extracts 12.5% of HMF into water using an organic/water mass ratio of 1:1 (Table 2). The extraction of HMF into water is much more voluminous when the CPME phase is contacting with several n-hexane, and 97% of the HMF can be extracted into water by this means.

### HMF production from disaccharides and polysaccharides

**Figure 5:**
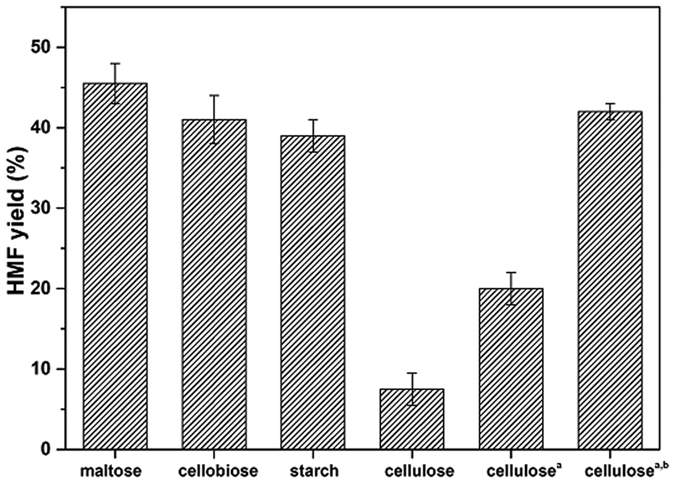
HMF yields from various carbohydrates in optimistic systems. Reaction conditions: carbohydrate = 0.55 mmol based on monosaccharide units, n (AlCl_3_) = 0.025 mmol, pH = 2.5, NaCl/aqueous phase mass ratio of 0.4, CPME to water volume ratio of 3, reaction temperature of 175 °C, reaction time of 20 min. ^a^Reaction temperature of 190 °C, reaction time of 60 min.

The optimized strategy was applied to the production of HMF from disaccharides (maltose and cellobiose) and polysaccharides (starch and cellulose) (Fig. 5). HMF yields from disaccharides were slightly lower than those obtained from glucose. Thus, our biphasic system is efficient in hydrolyzing the glycosidic linkage of disaccharides. Dehydration of starch to HMF was also quite efficient and selective (39% yield of HMF). However, only 5.5% yield of HMF was achieved from cellulose under the same conditions. The cellulose remained substantially unconverted as a solid suspension in the aqueous phase. According to the previous report^23^,^39^, this ineffective AlCl_3_-catalytic polysaccharides conversion is most likely due to keeping high crystallinity of cellulose at low temperature. When the conversion of cellulose was carried out at 190 °C for 60 min, the yield of HMF slightly increased to 19.5%. Cellulose was initially depolymerized to glucose, and then the glucose was dehydrated to HMF. There are at least two steps to convert cellulose to HMF. High crystallinity and polymerization degree in microcrystalline cellulose critically hinder the depolymerization process. To further increase the yield of HMF, cellulose was subjected to a pretreatment process prior to its catalytic conversion to HMF. To this end the crystallinity index of cellulose was decreased either by dissolution/regeneration in 85% H_3_PO_4_, after the pretreatments, cellulose was to be much less recalcitrant. During the catalytic conversion, cellulose was readily converted to HMF with 42.0% yield (Fig. 5). Although the additional energy contribution required by the pretreatment of cellulose by dissolution in H_3_PO_4_ nowadays represents a major drawback, coupling of our methodology with a recent work reported by Na^40^ and his coworkers in the field of hydrolysis of regenerated cellulose to water soluble sugars may open a more eco-efficient route for the production of HMF from cellulose.

### HMF production from corncob acid hydrolysis residues

**Figure 6:**
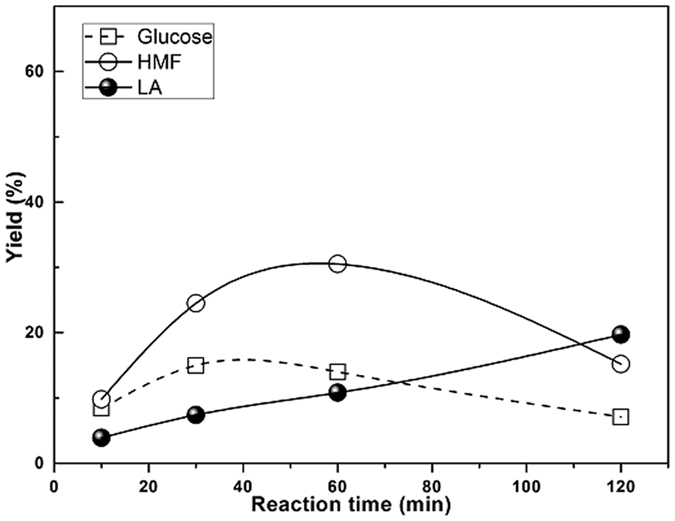
Yield to glucose, HMF and LA from CAHR. Reaction conditions: 5 wt % CAHR, n (AlCl_3_) = 0.025 mmol, pH = 2.5, NaCl/aqueous phase mass ratio of 0.4, organic to water volume ratio of 3, reaction temperature of 190 °C.

An efficient catalytic system for the synthesis of HMF from plant biomass as the feedstock can bring enormous benefits. Direct transformation of CAHR (a substantial pool of cellulose-enriched residues in China) into HMF was carried out. The effect of reaction time on the yield behavior and the product distribution is shown in Fig. 6. Molina *et al*.^28^ reported conversion of *Cynara cardunculus* into furans using H_2_SO_4_ as an acidic catalyst. Although the furfural yields were reasonable (75–95% based on the theoretical values), the HMF yields were very low (<10%). In our reaction system, the highest HMF yield of 30.5% was achieved after 60 min of reaction time. The result clearly demonstrated that AlCl_3_-HCl-NaCl-H_2_O/CPME system gave comparative HMF yield and the system was more effective at converting cellulose in CAHR. Furthermore, the process can reduce the step of production of glucose and the corresponding separation costs. Longer reaction times lead to more rehydration of HMF to LA, indicating that HMF was not stable at high reaction temperatures. Thus, to maximize overall HMF yield in the biphasic system, the reasonable reaction time of 60 min was chosen to preserve the target product. In this catalytic system, cellulose in CAHR seems to undergo acid-catalyzed hydrolysis through a generally tandem avenue^41^. Cellulose depolymerizes to glucose in acid medium. Glucose dehydrates to HMF due to the synergy between different catalytic sites. The rehydration of HMF results in LA formation. Besides, humins formation may be generated through self-polymerization of HMF or cross-polymerization between fructose and HMF.

## Conclusions

Herein, the use of AlCl_3_ and HCl as co-catalysts for the conversion of carbohydrates to 5-HMF was shown to be highly effective, allowing 5-HMF yields of up to 54.5%, and was effective on various disaccharides and polysaccharides. As AlCl_3_/HCl catalysts overcome equilibrium limitations of the glucose-fructose isomerization, the co-catalysts AlCl_3_/HCl could be superior to AlCl_3_ or HCl alone. CPME facilitated rapid glucose conversion and effective HMF separation from the aqueous phase containing catalysts and NaCl. Moreover, the aqueous phase containing catalysts and NaCl could be recycled for four times with minimal loss of activity. The highest HMF yield of 30.5% was directly obtained from CAHR. This approach presented here is a promising method for the production of HMF directly from biomass.

## Methods

### Materials

Glucose, fructose, cellobiose, maltose, starch, CPME, HMF, and LA were purchased from Aladdin Industrial Inc. (Shanghai, China). Cellulose was purchased from Sigma-Aldrich. AlCl_3_ and NaCl were purchased from Sinopharm Chemical Reagent Co. Ltd. (Shanghai, China). HCl (37 wt%) was obtained from Beijing Chemical Co. Ltd (Beijing, China) and used to adjust the pH value of solutions. All chemicals were used directly without further purification.

CAHR was kindly provided by Longlive bio-technology Co., Ltd. (Shandong, China), which was generated during the production of xylose. For experimental use, it was placed in the deionized water for 12 h, and washed with deionized water until neutral pH. The sample was then knife milled and passed through a 1 mm particle size interior sieve. All samples were dried in a vacuum drying oven at 50 °C overnight. The main composition of CAHR was as follows: 60.0 ± 0.5 wt% glucan, 6.0 ± 0.3 wt% xylan, 21.0 ± 0.2 wt% lignin according to the established National Renewable Energy Laboratory procedure^42^.

### General reaction procedure

All batch reactions were conducted in 15 mL thick-walled glass reactors. The aqueous layers containing 0.025 mmol AlCl_3_ were normalized to pH 2.50 by titrating with minute amounts of HCl. NaCl was also added to the pH-adjusted solution, and glucose was added to obtain a 10 wt% aqueous system. As a general procedure, 1.0 g of the aqueous feed and CPME was added to the reactor. Then the reactor was immersed in an oil bath at desired temperature under magnetic stirring (500 rpm) for a desired time. In a typical experiment for the conversion of other carbohydrates in a biphasic system, other carbohydrates (0.55 mmol based on monosaccharide units) were added in the reactors. In a typical experiment for the transformation of CAHR, 0.05 g of CAHR was added into the reactor. Upon completion of the reaction, reactors were cooled by nitrogen flow. Aqueous and organic phases were separated by extraction. Prior to analysis, samples were collected by filtering through a 0.22 μm syringe filter.

### Product analysis

The aqueous layer was analyzed by an Agilent 1260 HPLC system equipped with a Bio-Rad Aminex HPX-87H organic acid column and refractive index (RI) detector with an eluent (0.005 M sulfuric acid) flow rate of 0.6 mL min^−1^. The organic layer was determined using a Symmetryshield RP C18 column, a diode array detector, and an acetonitrile-water gradient at a flow rate of 1 mL min^−1^. The product was quantified by an external standard method based on the average peak area of each product.


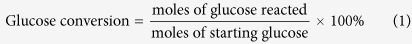



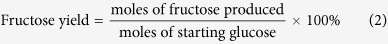



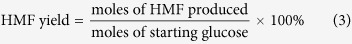



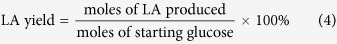



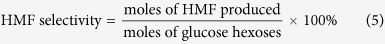


For other carbohydrates, HMF yields are defined as follows:


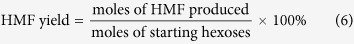


We elected to treat quantified CAHR hexosans as glucan. HMF production was the main focus of our work. Glucose and LA content were shown in the series of experiments. The yield of liquid products is defined as follows:


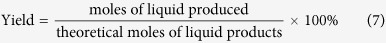


The pH of the aqueous phase (at 20 °C) was measured on a Sartorius PB-10 pH meter calibrated with standard buffer solutions.

## Additional Information

**How to cite this article**: Wang, C. *et al*. Synergy of Lewis and Brønsted acids on catalytic hydrothermal decomposition of carbohydrates and corncob acid hydrolysis residues to 5-hydroxymethylfurfural. *Sci. Rep.* **7**, 40908; doi: 10.1038/srep40908 (2017).

**Publisher's note:** Springer Nature remains neutral with regard to jurisdictional claims in published maps and institutional affiliations.

## Figures and Tables

**Table 1:** Glucose conversion with AlCl_3_ in different catalytic reaction system.

Entry	System^a^	Catalyst	Conv. (%)	Fruc. (%)	HMF (%)	LA (%)	HMF sel. (%)
1	H_2_O-NaCl/CPME	HCl^b^	15.5	ND^c^	4.5	1.5	29.0
2	H_2_O-NaCl/CPME	AlCl_3_^d^	92.5	13.0	46.0	6.5	49.5
3	H_2_O-NaCl/CPME	AlCl_3_/HCl^e^	89.5	7.5	54.5	8.0	61.0

^a^Reaction conditions: 10 wt% glucose in water (0.55 mmol), NaCl/aqueous phase mass ratio = 0.4, reaction temperature of 175 °C, reaction time of 20 min. ^b^A dilute HCl solution with pH adjusted to 2.50 was used. ^c^Not detected. ^d^0.025 mmol of AlCl_3_ was added in the aqueous phase. ^e^0.025 mmol of AlCl_3_ was normalized to pH value of 2.50 by titrating with minute amounts of HCl. Fruc: fructose. HMF sel: HMF selectivity.

**Table 2:** Extraction of HMF from CPME into water.

CPME^a^ (g)	H_2_O (g)	n-Hexane (g)	HMF extracted to water (wt%)
1.0	1.0	0	12.5
1.0	1.0	1.0	28.5
1.0	1.0	5.0	77.0
1.0	2.0	5.0	85.5
1.0	2.0	15.0	95.0.
1.0	3.0	10.0	97.0

^a^CPME solutions contacting 0.10 wt% HMF.
